# Distinguishing volumetric content from perceptual presence within a predictive processing framework

**DOI:** 10.1007/s11097-019-09632-7

**Published:** 2019-08-05

**Authors:** Sam Wilkinson

**Affiliations:** Department of Sociology, Philosophy and Anthropology, University of Exeter, Exeter, UK

**Keywords:** Predictive processing, Perceptual presence, Sensorimotor contingencies

## Abstract

I argue for an overlooked distinction between perceptual presence and volumetric content, and flesh it out in terms of predictive processing. Within the predictive processing framework we can distinguish between agent-active and object-active expectations. The former expectations account for perceptual presence, while the latter account for volumetric content. I then support this position with reference to how experiences of presence are created by virtual reality technologies, and end by reflecting on what this means for the relationship between sensorimotor enactivism and predictive processing.

## Sensorimotor contingencies and volumetric content

Consider the following, which we might call “the puzzle of volumetric content”. When I see a tomato, I typically see it as three-dimensional: a roughly spherical object with a rear surface that is occluded from view. How is it that my experience has this volumetric, three-dimensional content given that all that is actually presented to me are the surfaces of the tomato that are facing me?^1^ The account that Alva Noë (e.g. O’Regan and Noë 2001; Noë 2004) gives is that I have certain embodied expectations about how the visual sensorium would change if I were to act in certain ways. Crucially, these are expectations that are contingent on my actions (hence the term “sensori*motor* contingencies”). According to this account, without these embodied action-based expectations, I would not be able to experience the tomato as three-dimensional. Here is one of many passages where Noë presents this view.

Our perceptual sense of the tomato’s wholeness – of its volume and backside, and so forth – consists in our implicit understanding (our expectation) that movements of our body to the left or right, say, will bring further bits into view. Our relation to the unseen bits of the tomato is mediated by patterns of sensorimotor contingency (Noë 2004, p. 63).

It is certainly true that what I am presented with underdetermines the volumetric content of my experience. For example, a tomato that’s been hollowed out at the back would impact on my nervous system in the same way. Indeed, assuming monocular vision, a very realistic two-dimensional tomato façade could do the same. Furthermore, Noë is surely right that one promising way of building up from the sparseness of what is sensorially presented is through the notion of expectations (which reflect an “implicit understanding”). However, should these expectations be restricted to how the sensorium would change *if I were to act in certain ways*? Sure, one might argue, expectations about how the sensorium would change *if the object itself were to move in certain ways* (with me staying put) are as important, or indeed more so. Thus, if the tomato were spinning on a turntable, the expectation of what I’m about to see, as previously unseen parts of the tomato come into view, would be central to my experience of the tomato as a three-dimensional object. Furthermore, it seems that the cases where my expectations involve my acting are only tangentially to do with my action. They are, at least fundamentally, about the bringing of unseen parts *of the object* into view, regardless of whether this is about my moving around the object, or the object’s rotating on its own. Such a criticism has been put forward by Cavedon-Taylor (2011). In his words, Noë wrongly prioritizes “agent-active sensorimotor expectations”, when he should be prioritizing “object-active” ones.

In this paper, I want to show how a recent way of fleshing out Noë’s view in terms of predictive processing not only addresses Cavedon-Taylor’s criticisms, but also allows for a very neat distinction that Noë’s view cannot accommodate. This distinction is between *perceptual presence* on the one hand, and *volumetric content* on the other. These two things are unfortunately often conflated. An upshot of this is that predictive processing doesn’t simply flesh out Noë’s account: it accommodates its core insights and improves upon it.

## A predictive processing account of sensorimotor contingencies

Seth (2014) has re-cast sensorimotor contingencies in terms of the predictive processing framework (PPF) (Friston 2005; Clark 2013; Hohwy 2013). According to this framework, we perceive the world not in virtue of our nervous systems taking inputs from the world and constructing a percept, but in virtue of them generating models of the world, based on how well these models predict imminent sensory input. These models correspond to our experience of the world, and if they do a good job of predicting sensory input, they are kept. If not, and then they generate too much prediction error, they are updated or discarded. This is often put in terms of the brain being fundamentally in the business of minimizing prediction error.

On this framework, it is not just volumetric content that is underdetermined by sensory input. All perceptual experience, to the extent that it is determined by the model selected to predict future sensory input, is underdetermined by sensory input. Many predictive models are compatible with any given input. What helps with model selection in the face of this multiple compatibility is background knowledge about the probability of the model independently of the input, namely, its “prior probability”. (This is why the PPF is often seen as a fundamentally Bayesian approach.) Crucially, the predictive models that our nervous systems generate include ourselves as part of the world, and how our acting on and in the world will impact on our sensory (and proprioceptive) input.

Seth presents the connection between the PPF and sensorimotor contingencies as follows. The models that our nervous systems select, and which determine our experience, vary in their *counterfactual richness* (see also Friston et al. 2012). What he means by this is that my nervous system doesn’t simply have expectations about what is going to happen, namely, how the sensorium *will* change given what is likely to happen in the near future. There is also (at least in the human brain) a wealth of counterfactual expectation about how the sensorium *would* change given relevant circumstances – circumstances that needn’t happen and may even be unlikely to happen. In other words, my nervous system isn’t simply “content” with superficially predicting what will happen next, but seeks to “comprehend” more fully the statistical structure of the world, which involves, in part, the tacit positing of the underlying natures and dispositions of things, even if those natures and dispositions are never explicitly revealed, since their being revealed might involve an excessively complicated and/or unlikely set of counterfactual circumstances.^2^ This more costly enterprise clearly amounts to a wise long-term investment, since it prepares you for a causally complex and hard-to-predict world.

A crucial aspect of this picture, which I’ve briefly mentioned, is that predictive models aren’t just about the world, but include ourselves as part of that world. Indeed, bodily action is explained within this framework as a self-fulfilling prediction. More specifically, it involves the expectation that certain body-involving sensory and proprioceptive events will take place, which is then fulfilled by the ensuing action. In a way that is very much in keeping with Noë’s view, exploratory touch can be seen as a nicely illustrative case of perception and how it is fundamentally continuous with action: the movement of my arm and hand as I reach out in the dark to feel something is transparent since the expectations are being effortlessly fulfilled, and a model of the distal object is constructed as parts of the world with which I am less familiar, have less control over, are encountered and generate prediction error that needs to be accommodated.

With these two ingredients in place, namely, (i) the counterfactual depth of predictive models, and (ii) the deployment of predictive models that feature ourselves as part of the world, our nervous system has an implicit understanding of sensorimotor contingencies insofar as it deploys predictive models about how the sensorium will change if I were to act in certain ways (even if those ways of acting are unlikely).

As Seth puts it,

Extending this idea, counterfactually-rich generative models explicitly encode the conditional nature emphasized by the *mastery* of sensorimotor contingencies relevant to the puzzle of perceptual presence. That is, a counterfactually-rich hierarchical generative model explicitly encodes probabilistic representations of the external causes and expected values and precisions of the fictive sensations conditioned on repertoire of possible actions, thus capturing the key notion within sensorimotor theory of somehow perceiving parts of an object not directly available within ongoing sensory flux. (Seth 2014, p.106)

Seth goes on to use these ideas to explain why sensory experiences in synesthesia lack perceptual presence. In short, the story goes, it is because the sensations do not change in a coherent manner conditional on the subject’s actions, and therefore a real-world external cause is not posited by the nervous system. A similar story, of course, could be told about far less exotic phenomena, such as retinal afterimages, but the point is especially powerfully made in the case in synesthesia.^3^

## Volumetric content vs. perceptual presence

How can it be true, as I think it is, that we are perceptually aware, when we look at a tomato, of parts of the tomato which, strictly speaking, we do not perceive. *This is the puzzle of perceptual presence*. (Noë 2006, p. 414, emphasis added)

From this representative passage we can see that Noë thinks of, at least what he is calling, “the puzzle of perceptual presence” as the very same problem as the problem that I am calling “the puzzle of volumetric content”. But here is an importantly different puzzle, one which I think is more suited to being called “the puzzle of perceptual presence”. When I see a tomato, I don’t only see its facing surfaces, nor indeed do I only see it as three-dimensional; I also see it as *present*, namely, as part of the world that I inhabit. Again, sensory input underdetermines my experience of the tomato as present. I could after all experience it as an elaborate and ultra-realistic hallucination, an endogenous product of my brain that might, for example, stay in the same part of my visual field regardless of where I looked (like some sort of highly complicated and vivid after-image). In short, seeing something as part of my world is quite different to seeing it as three-dimensional, although both are underdetermined by current sensation. Where does *this* experience of presence come from?

Before answering this question, let’s clarify still further the relevant notion of perceptual presence and how it differs from, and can even come apart from, volumetric content. I can perceive a two-dimensional tomato façade as present, although I need not experience it as three-dimensional. Conversely, I might have as my screensaver an animation of a rotating tomato. There is an important sense in which I experience the tomato as three-dimensional, as having volumetric content, but, I do not thereby experience it as having presence (I do however experience the monitor it’s on as having perceptual presence).

I want to suggest a very simple solution to both puzzles that falls quite naturally out of the PPF. Borrowing Cavedon-Taylor’s (2011) distinction between object-active and agent-active expectations, I want to say the following. *Whereas object-active expectations generate volumetric content (experiences of three-dimensionality), agent-active expectations generate perceptual presence (experiences of things as part of my world)*. On this account, the experience of the screensaver tomato has volumetric content because I have certain (in this case accurate) object-active expectations about how the sensorium will change as the virtual tomato rotates. However, it lacks perceptual presence because I don’t have the relevant agent-active expectations about the tomato. In fact, I have expectations that contradict presence, e.g., that if I were to walk around it (or rather where it is represented as being), all I would see is, first, an unrealistic foreshortening of the tomato from round to oblong as I see my computer screen from an angle, and then, as I continue round, the back on my monitor. While the *tomato on the screen* is experienced as having volumetric content but not perceptual presence, the *monitor itself* of course is experienced as having both.

At this point I’d like to clarify object-active expectations still further, by in particular clarifying their relationship to action.^4^ In the wild, so to speak, three-dimensional inanimate objects tend not to spontaneously rotate. Our exposure to the occluded surfaces of objects is usually through our action, e.g. walking around the object. This observation in no way contradicts my point about object-active expectations, since this in not about how these expectations are acquired, but about what they fundamentally are once acquired, namely, expectations generated by models about the object. As mobile, embodied agents it makes perfect sense that these object-active expectations, and ultimately our generative models for objects, should be acquired through exploratory action. Once acquired, however, they pertain to the object, not to our actions in relation to the object. This is indeed why, even if ecologically unusual, experiencing, say, an apple as three-dimensional rather than flat, or indeed whole rather than with a bite out of the back, includes expectations about how the sensorium would change if the apple itself were to rotate and we stood still. Our volumetric appreciation of the apple is at play when occluded surfaces come into view without surprise, regardless of whether this coming into view is achieved by walking around the object, or the object itself rotating. The difference between these two modes of bringing our volumetric appreciation into play is that the former, action-involving one also necessarily involves an appreciation of presence, whereas the latter can involve presence but needn’t (as the rotating screensaver illustrates).

This under-appreciated distinction between volumetric content and perceptual presence, and the different kinds of expectations that underpin the two, offers Noe a way out of Cavedon-Taylor’s criticism: Noe was talking about perceptual presence, not about volumetric content, and it is the former, and not the latter, that is underpinned by sensorimotor contingencies. However, this is unsatisfying for two reasons. First, it is not clear that Noe is restricting himself to perceptual presence in this more specific sense (see the passage at the start of this section). Second, even if we grant that he is, surely we want to be able to accommodate both perceptual presence and volumetric content? I now present account that does just that.

## Beyond sensorimotor contingencies

It is crucial to see that not all counterfactual predictions are about what would happen were *I* to *do* something. Many of the relevant expectations are to do with what will happen to the object in certain circumstances that need not involve my acting at all (although of course they may). So here we get the distinction between object-active and agent-active expectations. These expectations, although they are responsible for generating volumetric content and perceptual presence respectively, in being responsible for all experiential content, go well beyond that. They may, for example, generate the experience that something is fragile (It would smash if dropped; an object-active expectation) or edible (I could pick it up and eat it; an agent-active affordance-based expectation) and so on.

So what story does this framework generate for volumetric content and perceptual presence respectively? Let’s return to the rotating tomato screensaver. As the tomato rotates, bringing the back of the tomato into view, the model that my nervous system adopts in order to best predict imminent inputs is that there is an object with a certain shape and volume occupying Euclidean space.^5^ There is no need, at least when we’re purely considering volumetric content, to model myself or my potential actions.

For perceptual presence – for example, with a real tomato on the table in front of me – my nervous system selects a model that underpins my embodied appreciation (my “implicit understanding”) that, among other things, as I move around the tomato, previously unseen parts of the tomato will come into view. In short, my nervous system has generated the optimally predictive model that there is an object *there* in relation to *me.* It is, like me and my body, a part of my world, the world that I can navigate through and act upon.

So, to sum up, then, within the PPF we are to understand the nervous system as fundamentally engaged in the selection of optimally predictive models, and these models determine the content of our experience at a given time. When our experience contains a three-dimensional object, that three-dimensionality (taken, for theoretical purposes, in isolation) is best explained in terms of our nervous system having selected a model *for the object*; it models the object to the extent that it exploits object-centred counterfactual expectations. That very same experience may (and usually will) also have *presence* in its content, namely, the object is experienced as part of my world. This quite different feature of the experience is best explained in terms of my nervous system having selected a model for the overall encounter, namely, for *the object in relation to me*; it exploits agent-centred counterfactual expectations, namely, sensori*motor* contingencies. The relevant expectations concern how the sensorium will change if I were to act in certain ways (even if I am highly unlikely, or even unable, to do so).

## Creating presence in virtual reality

One way of putting this account to the test is to examine the ways in which experiences of presence can be created with virtual reality (VR) technologies. In VR research, presence refers to the extent to which a virtual (i.e. non-actual) environment (VE) is experienced as an actual environment that the subject is spatiotemporally located within. The VE usually takes the form of a computer-generated pictorial representation presented to the subject via a head-mounted display (HMD). The ambiguity in the word “presence” of what it is that is present turns out to be rather apt. That is because it refers simultaneously to the presence *of the subject* in the environment, and the presence *of the environment* to the subject. In short, presence is a two-way relation between subject and environment. If I experience the environment as present to me, I thereby experience myself as present in the environment.

It has long been acknowledged that what generates experiences of presence in VR is sensorimotor contingencies (viz. agent-active expectations) (Slater and Wilbur 1997). For example, where the VE involves the subject standing in front of a precipice, the better the calibration (the better the coherence and the smaller the delay) between head movement and visual display updating, the greater the experience of presence (as measured by increased heart rate) (Meehan et al. 2003). Furthermore, agent-active expectation is not merely one of a number of ingredients, which, other things being equal, increases the experience of presence. It seems to be the main determinant of experienced presence. For example, the visual realism of the display plays only an enabling role (viz. it is only relevant to the extent that it can support sensorimotor contingencies). To illustrate this point, if you compare a very realistic, high-resolution, three-dimensional environment presented from a first-person perspective that you navigate with a joystick or handheld controller, with a relatively unrealistic, low-resolution environment that you navigate by moving your head, so that the scene pans across your visual field from left to right as you move your head from right to left, and vice versa, it is the latter, which, in spite of its low resolution, is more “immersive”, namely, generates a far stronger experience of presence. Not only this, but in cases where the sensorimotor contingencies are generated and the only variable is the realism and resolution of the visual display, experienced presence doesn’t seem to increase in line with better resolution (for example, there was no rise in the rate of heart-rate increase depending of whether the precipice was realistic or not (Zimmons and Panter 2003)). In other words, once the visual display crosses a minimal threshold where it enables sensorimotor contingencies, any further increases in realism or resolution are superfluous, at least as far as generating the experience of presence is concerned.

Much more effective in enhancing presence are the addition of subtle movements of the virtual visual field that mimic the movements of walking, and are calibrated to the subject’s actual walking (Slater et al. 1995). Another obvious sensory consequence of action is involved in the perception of one’s own body, e.g. when you lift your hand in front of your face and look at it. It has been shown that experienced presence can be greatly enhanced if, in addition to how the view of the environment changes as one moves (e.g. one’s head) one also adds a representation of the subject’s body, and especially limbs, that move in line with the subject’s actual limb movement (Slater and Usoh 1994). For example, the subject sees an arm in front of her face when she actually lifts her arm in front of her face (and the virtual arm might then rotate as the subject rotates her arm). This clearly requires the VR system to detect and virtually reproduce the subject’s actual movement (something VR technology has been able to do for some time). This further enhancement of presence supports the close connection between presence, action and embodiment. It also gets very simply and effectively accounted for within the PPF. For example, as I hold my hand in front of my face and the HMD shows me a hand (which can deviate in appearance quite considerably from my actual hand) I might then rotate my hand (e.g. to show myself the back instead of the palm), and if the display is well calibrated, I will experience the hand both as present and three-dimensional (and as mine).

So much for VR and presence; what about volumetric content? If my suggestion is correct, VR is not necessary for experiences of volumetric content, since what the HMD provides is exploitation of agent-active expectations, not the object-active ones that underpin three-dimensionality. This seems, on reflection, to be an accurate supposition. Rudimentary experiences of three-dimensionality can be achieved through perspective (think of a *trompe-l’oeil*) and, as discussed, rotation. More sophisticated and immersive forms of three-dimensionality exploit the depth cues that binocular vision provides (see fn. 1), viz., 3D glasses give each eye a slightly different image, namely, the image as it would be from a slightly different location, namely from the location of each eye. This means that a relatively large disparity between the two images corresponds to experiences of proximity, whereas a relatively small disparity corresponds to experiences of distance. This does not exploit action. You get this effect while sitting perfectly still. Indeed, moving your head actually disturbs the effect of 3D glasses since the depth cues don’t vary as they ought to. Having said this, if they did, and you combined VR and 3D, it would add fuller presence to the experience.

Again, a point of clarification is required here. 3D glasses do generate illusions of presence as well as three-dimensionality, and the urge to reach out and touch the Tyrannosaurus Rex (for example) is testament to that. The illusion, however, is fragile, and movement destroys it rather than strengthening it. But this is perfectly in keeping with the distinction I am trying to sketch. Agent-active expectations are invoked by the careful recreation of embodied binocular vision that 3D glasses (and the appropriate image) provide. But these expectations are broken when movement doesn’t generate the expected sensory change. At the same time, of course, object-active expectations are also broken yielding a loss of volumetric content, as discussed. As the T-Rex’s head appears to jut out of the screen (let’s suppose head-on), if you try (by moving) to look at it from the side, your nervous system will be disappointed, and your 3D generative model of the T-Rex (as a 3D object) will collapse, since the image projected on the cinema screen won’t change to show you the dinosaur in profile simply for your benefit (it will also, more subtly but beforehand, fail to retain the carefully recreated binocular disparity that 3D technology exploits).

## Sensorimotor Enactivism and predictive processing

In spite of appearances, I don’t think that what I have said here is bad news for Noë’s sensorimotor enactivism. Noë’s view is that our conscious perceptual experience is determined by sensorimotor contingencies, by what I have here called agent-active expectations. To the extent that our perceptual experience is about perceiving the world around us and perceiving it *as* around us, what I have said leaves the core of Noë’s sensorimotor enactivism intact. In that respect it’s a defense of Noë against the criticism put forward by Cavedon-Taylor, but one that elucidates the notion of presence. And although in theory Cavedon-Taylor is right that it isn’t agent-active expectations that generate volumetric content, in practice it is hard to separate volumetric content from presence in an experience that is to be genuinely thought of as *perceptual* (after all, I don’t literally perceive a tomato in the screensaver example: I perceive the monitor and its screen). Perhaps more importantly, it is hard to conceive of a conscious, embodied organism that wasn’t constantly making use of sensorimotor expectations to at least some extent. In a related manner, when I talk about volumetric content independently of perceptual presence, this is a theoretical abstraction that never occurs in practice, at least not for embodied organisms (and these are the beings in the universe that perceive and have need to do so). It is nevertheless a theoretical distinction that is worth making since it points to two different components of experience that can be manipulated separately (albeit in unecological circumstances). Certain vivid after-images, or rotating screensavers, might have relatively high levels of three-dimensionality, but low levels of presence. Some low-resolution VR experience might have the opposite: high presence, but low three-dimensionality.

Finally, I’d like to gesture towards two ways in which sensorimotor enactivism and predictive processing are not only compatible, but also deeply similar in spirit. The (related) things that they share (and there a likely more) are as follows.

First, both the PPF and sensorimotor enactivism explicitly think of perception as, to quote Noë, “a thoughtful activity.” It’s not about passively receiving “impressions” of the world, but of actively *making sense* of the world. Second, both the PPF and sensorimotor enactivism, when compared to more orthodox views of perception, play down the importance of sensory stimulation for perceptual experience. To clarify, both entail that my perceptual experience at t1 is not determined by (or worse, constructed out of) antecedent sensory stimulation. It is rather, for both views, determined by patterns of expectation, which may be fulfilled or frustrated. The role of sensation for both views is always as a corrective after the experience itself, which is profoundly dynamic. Of course, these expectations are cashed out within the PPF as “models” or “hypotheses”, which is a move sensorimotor enactivists are likely to resist, but that is a question for another day (see Downey 2018).

## Conclusion

In this paper I have presented a distinction between perceptual presence, on the one hand, and volumetric content, on the other, and fleshed it out in terms of predictive processing. Whereas presence is underpinned by what Cavedon-Taylor (2011) calls agent-active expectations, volumetric content is underpinned by object-active expectations. Within a predictive processing framework, the former expectations are generated by a predictive model that centrally involves the agent and their relationship to the object (or environment more generally). The latter expectations, in contrast, are generated by models of the object, by how the agent’s nervous system takes the object to be, regardless of its actual, real location in relation to the agent. Although this model of the object may have been constructed in a way that makes use of exploratory action, the model itself does not centrally involve action in the way that it does for perceptual presence. I have supported this distinction by using a number of examples, including examples from VR, and, finally, reflected on commonalities between sensorimotor enactivism and predictive processing.

## Footnotes

1 Of course, there are effects of three-dimensionality generated by binocular vision, but since monocular vision also generates experiences of objects as three-dimensional, we will put the added benefits of having two eyes to one side.

2 The scare quotes around “content” and “comprehend” are meant to signal that this is metaphorical. Only entire organisms are literally capable of being content and of comprehending things – not nervous systems. I feel that the utility of these non-literal ways of speaking far outweighs any confusion that may arise.

3 Ward (2012) gives a nice explanation, in terms of sensorimotor contingencies, of why synaesthetic colours don’t adapt away. This account could be very nicely fleshed out with predictive processing.

4 I would like to thank an anonymous referee for prompting me to make this clarification.

5 Note that a rotating cube works better than something spherical (since sensory inputs change more drastically contingent on the rotation of a cube than of a sphere – indeed a perfect sphere that is uniformly coloured ought not to be subject to detectable rotation). Note also that the ambiguous Necker cube gets a very neat treatment here: the two working hypotheses (namely, where the left-most square is seen as either the outer front side, or the inner back side) entail different counterfactual predictions contingent on motion of the (hypothetical) cube.
